# Trismus Nascentium

**Published:** 1871-01

**Authors:** Theo. W. Stull

**Affiliations:** Marengo, Ill.


ARTICLE V. — *Trismus Nascentium.* By THEO. W. STULL, M. D., Marengo, Ill.

In reporting the following case, it is not with the fullest confidence that the recovery is attributable to the remedies used, and yet I am so fully persuaded of the efficiency of the particular remedies on which I finally relied, that I should most certainly resort to a like treatment in a similar case.

On the 8th of March last I was called to see a child of Mr. J. L., which he said was nine days old, and seemed to have a sore mouth, as it cried and would not nurse. I found a good reason for its not nursing in the closed jaws, and muscular rigidity.

Upon examination I found an erysipelatous blush extending an inch or an inch and a half around the umbilicus, from which a little unhealthy pus was oozing. The prescription was, Pot. Bromide, gr. iss, F. E. Aconite, gtt. ss, in solution, every two hours, and also the application of Carbolic Oil to the umbilicus, with continual fomentation. To be fed the breast milk with a spoon, though it swallowed with difficulty. Under this treatment the jaws relaxed considerably, and yet not being quite satisfactory, various other remedies were resorted to and abandoned, all now superceded by the original prescription, which was now persevered in to a somewhat tardy convalescence. An occasional warm bath was added to the original treatment in place of the fomentation.

I remark that these were usually cleanly people, though in poor circumstances. And various as the theories are as to the cause of Trismus, I am fully persuaded that in this case, at least, the cause was phlebitis of the umbilical veins.

---

***Traveling.***

The best mode of travel is on foot or on a saddle; the next best is in an open carriage, or in an old-fashioned stage coach. A sea voyage, and almost any mode of traveling by water, is, in general, useful; but it would be a serious practical joke if any one were to advise an invalid to seek for health in a railroad car.—*Medical Bulletin.*

